# Gross on Urinary Diseases, Etc.

**Published:** 1852-09

**Authors:** 


*Gross on Urinary Diseases, etc.* — Medical and other writers of our country have frequently complained of a disposition on the part of British critics to disparage productions emanating on this side of the Atlantic. If there are just grounds for such complaints, the signal favor with which the late work by Prof. Gross, of the University of Louisville, has been received, is some indemnification for former acts of injustice. The British and Foreign Medico-Chirurgical Review, in speaking of the admirable treatise on Diseases of the Genito-urinary system, a treatise of which the American medical profession may justly be proud, holds the following language: — "Dr. Gross is quite correct in asserting that all the treatises on this subject as yet published in the English language, are mere outlines, which no one has attempted to render at all worthy companions to such works as those of Lawrence on 'Hernia,' Mackenzie on the 'Diseases of the Eye,' Budd on the 'Liver,' and Curling on the 'Testis.' It has remained for an American writer to wipe away this reproach; and so completely has the task been fulfilled, that we venture to predict for Dr. Gross's treatise a permanent place in the literature of surgery, worthy to rank with the best works of the present age. Not merely is the matter good, but the getting up of the volume is most creditable to transatlantic enterprise; the paper and print would do credit to a first-rate London establishment; and the numerous wood cuts which illustrate it, demonstrate that America is making rapid advances in this department of art. We have, indeed, unfeigned pleasure in congratulating all concerned in this publication, on the result of their labors; and experience a feeling something like what might animate a long expectant husbandman, who, oftentimes disappointed by the produce of a favorite field, is at last agreeably surprised by a stately crop which may bear comparison with any of its former rivals. The grounds of our high appreciation of the work will be obvious as we proceed; and we doubt not that the present facilities for obtaining American books will induce many of our readers to verify our recommendation by their own perusal of it."*

Again, in comparing the work by Dr. Gross with a treatise on similar subjects by an English writer, Coulson, the reviewer adds: — "We have already expressed our high appreciation of Professor Gross's treatise; and it gives us much regret to feel compelled to state that the comparison of the two is, on almost every point, disadvantageous to Mr. Coulson. We acknowledge that we have tried his work by a high standard; but we think that we are fully justified in so doing, considering how long its author has been before the public as a surgical writer, and how high a position he evidently desires to attain. There is scarcely any point on which his work is not inferior to Dr. Gross's; and we have been continually led to feel, in our examination of it, how seriously defective it is, alike in method and in completeness; so that its perusal has left us with the conviction, that scarcely any one subject has been satisfactorily treated, scarcely any point of pathology or practice fairly and fully disposed of. We can assure Mr. Coulson that we state this with regret, and with no other motive than that which arises from our desire to discharge our critical duty faithfully and impartially. That our verdict will be confirmed by any competent judge, who may take the same pains that we have done in the comparison of the two works before us, we have not the smallest doubt; and we have even a sufficiently good opinion of Mr. Coulson's own candor, to believe, that if he will examine Professor Gross's treatise for himself, he will admit our case to be so strong, that he will not feel it necessary to impute to us any bias to his disadvantage."

---

*Curators of Med. Department of University of Buffalo.* — We are requested to state that the names of the curators of the Med. Dept. of the University of Buffalo, residing in this city, were inadvertently left out of the list of curators in the annual circular for 1852. The names of the gentlemen referred to, are as follows: — Drs. E. Wallis, Samuel Carey, S. F. Mixer, and J. B. Samo.

---

* Not having read the proof of the eclectic department, it had escaped us that this extract is already copied (page 242.) It will, however, bear repetition.—Ed.

